# Development of a New Strategy for Studying the Oxygen Consumption Potential of Wine through the Grape Extract Evaluation

**DOI:** 10.3390/foods11131961

**Published:** 2022-07-01

**Authors:** Marioli Carrasco-Quiroz, Ana María Martínez-Gil, Ignacio Nevares, Víctor Martínez-Martínez, Rosario Sánchez-Gómez, Maria del Alamo-Sanza

**Affiliations:** 1 Department of Analytical Chemistry, UVaMOX—Universidad de Valladolid, 34004 Palencia, Spain; mariolialejandra.carrasco@alumnos.uva.es (M.C.-Q.); anamaria.martinez.gil@uva.es (A.M.M.-G.); rosario.sanchez@uva.es (R.S.-G.); 2 Department of Agroforestry Engineering, UVaMOX—Universidad de Valladolid, 34004 Palencia, Spain; victor.martinez.martinez@ui1.es; 3 Faculty of Science and Technology, Isabel I University, 09003 Burgos, Spain

**Keywords:** grapes, dissolved oxygen, oxygen consumption kinetics, red wine components, reconstitution, shelf life

## Abstract

The development of a method to determine the aging potential of wine at the time of harvest, through the evaluation of its oxygen avidity, is a potential tool for the winemaking sector. To this end, it is necessary to formulate a potential wine with this grape prior to alcoholic fermentation. The main objective of this method was to optimize a formulation of the potential wine, based on the grape extracts (GEs), to subsequently evaluate its oxygen consumption kinetics, guaranteeing maximum differentiation between the different GEs. The optimization was carried out with a Taguchi orthogonal matrix design, which optimized the variables to be used in the GE reconstitution. The variables studied were pH, Fe^2+^, Cu^2+^, Mn^2+^, alcohol content and acetaldehyde. The evaluation of the characteristic parameters of the consumption kinetics of each of the GEs allowed us to know the different reconstitution conditions that most influence the differentiation of the oxygen consumption kinetics of very similar GEs. The reconstitution conditions chosen were pH 3.3; 1 mg/L Fe^2+^; 0.1 mg/L Cu^2+^; 1 mg/L Mn^2+^; 12% (*v*/*v*) alcoholic strength and 10 mg/L acetaldehyde, with pH, Fe^2+^ and Mn^2+^ being the significant conditions. The kinetics of reconstituted GE could be a tool for the classification and evaluation of grapes according to their aging potential or shelf life of the wine made.

## 1. Introduction

Knowledge regarding the precursors of the grapes, in order to predict the characteristics of the wine to be produced, is of great interest in the winemaking processes, since it allows the winemaker to correctly manage the process and obtain maximum potential from the grapes [1,2,3]. The study of the potential of grapes can be carried out by reconstituting their phenolic and aromatic fractions in a model wine to study the potential characteristics of the final wine [2]. Thus, this methodology has been used to study the aromatic and phenolic potential of the final wine [1,2,3]. An aspect of great interest for the winemaker is understanding the effect of exposure to different levels of oxygen has on the wine, since wine consumes oxygen and the processes that take place are key to define its aromatic, sensory and taste characteristics [4,5,6]. Several studies have been carried out to determine the oxygen consumption capacity of wine by saturating it with air [4,5,6,7,8,9,10,11,12,13,14]. However, there is great diversity in wine saturation protocols, in oxygen consumption monitoring and the analysis of the obtained information.

The recently published work by del Alamo-Sanza et al. [15] highlighted the importance of the correct measurement of dissolved oxygen in a wine during its air saturation to properly monitor the kinetics of oxygen consumption. It also showed that the level of dissolved oxygen reached by a wine saturated with air depends on the type of wine, as well as on the environmental conditions (temperature, RH, and pressure). That work proposes a methodology for wine saturation with air that allows the kinetics of O_2_ consumption to be characterized and a series of parameters to be extracted which define and compare different types of wines. The two parameters that most differentiate the wines are the concentration of dissolved oxygen at half the time of consumption (O_mid_) and the time required to consume 90% to 10% of the dissolved oxygen initially available (∆t_0_90_10_).

In general, it has been shown that red wines have different O_2_ consumption kinetics from white and rosé wines, with a very high initial O_2_ consumption rate that varies according to the type of wine and the metals, especially Cu^2+^ and Fe^2+^ content, as indicated by other authors [10,16,17]. Nevares et al. [13] showed a correlation between the oxygen consumption characteristics of a wine and some chemical compounds, particularly copper and iron. Thus, wines with a higher copper content showed a higher rate of oxygen consumption, a result later corroborated by Carrascón et al. [7]. There are also reactions that are catalyzed by metals, such as Fe^3+^ ions that rapidly oxidize catechol [9], and it has also been shown that Mn^2+^ accelerates the oxidation of Fe^2+^ by increasing the rate of catechol oxidation in a model wine [18]. The rate of oxygen consumption has been found to increase when Fe^2+^ and Cu^2+^ concentrations increase in wine, so the catalytic activity of Mn^2+^ appears to be dependent on those concentrations [18]. Acetaldehyde is the most abundant aldehyde compound in wine. Its presence in small amounts has several implications in wine, as it can react with tannins and anthocyanins promoting the formation of stable color adducts [19,20,21]. The pH of wine plays an important role in its stability, so those with a high pH tend to overoxidize easily [22], while those with a lower pH have oxidation restrictions [23,24,25]. However, there is great variability in the results between the parameters that measure oxygen consumption and the chemical components of wines. Nevares et al. [13] demonstrated that it is not enough to know individualized chemicals to define the oxygen consumption rate of wines; it is necessary to evaluate the chemical properties of wines. All this demonstrates the importance of considering all the components as well as the concentrations for each of them.

Oxygen is present throughout the vinification, aging and storage process. During the maceration stage, oxygen allows a greater extraction of phenolic compounds, but can cause phenolic compound oxidation phenomena, browning or formation of highly reactive species, such as quinones, radicals and hydrogen peroxide, which act under the catalytic action of metals such as Cu^2+^ and Fe^2+^ [26]. Ferreira et al. [10] developed a partial least squares (PLS) regression model to explain the rate of oxygen consumption and its relationship with some phenolic compounds.

There is a consensus in the literature that oxygen management during winemaking determines the properties of the wine. Knowing the capacity that a wine may have to consume oxygen from the study of grapes has not been studied yet (there is no literature). This information would be of great interest to the oenologist since it would allow him to properly manage the vinification. The aim of this work was to optimize a method for grapes. For this purpose, the extracts of different grapes were reconstituted with different reconstitution conditions looking for the maximum differentiation in oxygen consumption kinetics between the different GEs. Thus, this is a new tool for the evaluation of the oxygen consumption kinetics of grapes, and for classifying them according to their aging capacity or shelf life.

## 2. Materials and Methods

### 2.1. Grape Extracts (GEs)

The three monovarietal grape extracts were prepared from two different grape varieties (2017 vintage), two Tempranillo (GE-A and GE-B) and one Garnacha (GE-C), supplied by Laboratorio de Análisis del Aroma y Enología (LAAE), University of Zaragoza (Spain) and obtained using the method indicated in Alegre, Arias-Pérez et al. [1]. Briefly, 10 kg of grapes were destemmed and crushed in the presence of 15% (*v*/*v*) ethanol and 5 g/hL of potassium metabisulfite (Merck, Germany), macerated for 7 days at 13 °C, then pressed, filtered, and stored at 5 °C in the dark. Subsequently, this resulting ethanolic must was dealcoholized in a rotary evaporator system (Buchi R-215 equipped with a V-700 vacuum pump from Buchi, Flawil, Switzerland) which was subsequently passed through a prepared 10 g C18 cartridge previously conditioned with 44 mL of methanol followed by 44 mL of milli-Q water with 2% ethanol. The cartridges were then washed with 88 mL milli-Q water at pH 3.5 and dried by allowing air to pass through them. Reconstituted grape extracts (GEws) were recovered by elution with 100 mL of ethanol.

### 2.2. Taguchi Experimental Design to Optimize GEs Reconstitution and Statistical Analysis

The oxygen consumption capacity evaluation method allows the differentiation of different grape extracts. The future wines have compounds that grapes do not have and, therefore, it is necessary to obtain a wine from the reconstitution of the grapes. The grape extract reconstitution is necessary beforehand and must be performed ensuring maximum differentiation of the GEs by its consumption kinetics. It is very important to note that the objective is to establish a reconstitution method that maximizes the differentiation of GEs produced from grapes with different characteristics. Parameters can vary according to the type of winemaking, and they were studied to see their effect on the kinetics of consumption. The advantage of this Taguchi design of experiments is that it allows the study of different variables simultaneously to achieve an objective. Moreover, it is not necessary to experiment with the levels of all factors, as the Taguchi orthogonal design indicates the combination of factors and levels necessary to study the different variables simultaneously. In this case, the objective is to obtain the GE reconstitution conditions that allow the greatest differentiation in the consumption kinetics. The Taguchi methodology was chosen for the experimental design, choosing different parameters in a possible concentration range in the wines produced. Each parameter was assessed at two different levels resulting in an L16 (2^15^) Taguchi orthogonal array. The experimental conditions are summarized in Table 1. The input parameters were pH: 3.3 and 3.9; Fe^2+^: 1 and 8 mg/L; Cu^2+^: 0.1 and 0.8 mg/L; Mn^2+^: 1 and 4 mg/L; alcoholic strength: 12% and 15% (*v*/*v*); and acetaldehyde: 10 and 30 mg/L. For Fe^2+^, Cu^2+^, Mn^2+^ and acetaldehyde dilutions of an iron (II) chloride 4-hydrate pure, copper (II) chloride 2-hydratemanganese (II) chloride 4-hydrate (all from Panreac-AppliChem, Castellar del Vallès, Barcelona, Spain) and acetaldehyde (>99.9%, Fluka, Madrid, Spain), respectively, were prepared and added to reach the previously indicated concentrations. The total acidity in all of them was 5 g/L of tartaric acid L(+)-Tartaric acid, reagent grade, Scharlab, S.L., Barcelona, (Spain) and a sodium hydroxide solution, Labbox Labware, S.L., Barcelona, (Spain) was used to adjust the pH.

Each parameter was assessed at two different levels resulting in a L16 (2^15^) Taguchi orthogonal array. The experimental conditions are summarized in Table 1. For each GE, 25 mL was prepared according to the sixteen different conditions. Once the GEs were re-constituted and thenceforth called GEws, 5 mL was destined for analysis and the other 20 mL for the saturation process in order to study the kinetics of oxygen consumption.

The 16 runs in the design matrix were randomly made for the 3 GEw studied, producing a total of 48 runs. Each of the GEws in each of the conditions was subjected to the oxygen consumption process, and the kinetic parameters were obtained. The 11 parameters of oxygen consumption kinetics established by del Alamo-Sanza et al. [15], which are described in the next section, were studied by an analysis of variance (ANOVA) test according Taguchi data treatment to determine which reconstitution conditions allowed the greatest differences between the parameters of the consumption kinetics of the 3 types of GEw. Therefore, for each of the 16 experiments, the *p*-level operator for each combination of the 3 studied GEw was obtained: thus, one *p*-level for the comparison between the first and the second GEw, another *p*-level for the second and the third GEw, and another *p*-level for the first and the third GEw. Each *p*-level indicates the statistical significance of the differences between the two GEws compared. Finally, a global aptitude indicator for the three GEw was obtained as the maximum *p*-value for the three pairs analyzed, meaning that the worst case of the one-on-one comparison was taken as the three GEw aptitude indicator. Therefore, the objective was to find the lowest aptitude in the comparative of the responses (parameters of oxygen consumption kinetics).

ANOVA according to LSD test (*p* < 0.05) and Pearson’s correlation analysis were conducted using the Statgraphics Centurion statistical program (version 18.1.12; StatPoint, Inc., Warrenton, VA, USA).

### 2.3. Kinetics of Oxygen Consumption

#### 2.3.1. Air Saturation of GEws

The 20 mL of each GEw were tempered to 35 °C following the method of Nevares et al. (2017) based on the Arrhenius equation, where increasing the temperature increases the rate of the reaction, and were then air-saturated according to del Alamo-Sanza et al. [15]. To prevent oxygen oversaturation in the equilibrated solution, high-speed air flow (i.e., air flow rates > 1 mL/min) and very small bubbles were avoided, as described by Näykki et al. [27].

#### 2.3.2. Measurement of Oxygen Kinetics Consumption

The oxygen-saturated GEw were then transferred into airtight 3 mL glass SensorVial SV-PSt5 (PreSens Precision Sensing GmbH, Regensburg, Germany). Reader and the oxygen consumption kinetic was monitored by measuring the dissolved oxygen (DO) employing this device.

Five replicates were performed for each one of the 16 experimental conditions and for each type of oxygen-saturated GEw. To ensure that all samples were measured simultaneously in the same conditions, the device with the samples was kept in a high-accuracy thermostatic chamber at a constant temperature of 35 ± 0.10 °C (Raypa Trade, Barcelona, Spain) in darkness. The DO of each sample was measured every hour throughout the consumption process, giving rise to a total of 240 oxygen consumption kinetics (48 × 5). The initial atmospheric pressure of each trial was checked with the digital barometer (Fibox-4 Trace device, PreSens GmbH, Germany) during every assay.

The oxygen sensors of each vial were calibrated according to the manufacturers’ protocol, with measurements performed at two calibration points: oxygen-free water at a concentration of 0 mg/L (0% air saturation) and saturated air (100% air saturation). Since the working temperature was fixed at 35 °C, this was considered in the calibration procedure.

#### 2.3.3. Kinetic Curve Data Process

To study the oxygen consumption kinetics, the curve data were preprocessed according to del Alamo-Sanza et al. [15] in order to obtain representative curves for each sample. To this end, each kinetic curve was preprocessed removing the initial and final data, that is, the data before the maximum and after the minimum of the curve, respectively. Samples before the maximum were removed because they were acquired before the consumption process started, while the minimum of the curve was considered as the end of the consumption process, so data after that was removed as it was not representative of the consumption kinetics. The curves were then resampled with a sampling period of 15 min and combined, obtaining the mean − std and mean + std curves of the five repetitions of the kinetic curves of each sample. As a result, the 240 curves obtained from the 48 samples analyzed were reduced to a total of 96 curves (32 for each type of GEws).

The 11 selected parameters were as follows: total oxygen consumed (hPa) ∆*O_max_min_* = *O_max_ − O_min_*; variation between oxygen 90% and oxygen 10% (hPa) as ∆*O*_90_10_ = *O*_90_ − *O*_10_; oxygen value that represents 10% of the range between the maximum and minimum values (hPa) as *O*_10_ = *O_min_* + 0.1 (*O_max_ *−* O_min_*); oxygen at half consumption time (hPa) as *O_mid_ = O*_2_ (*t *=* t_Omin_*/2); minimum/final oxygen value (hPa) as *O_min_ *=* min *[*O*_2_(*t*)]; area under the oxygen consumption curve (hPa·h) as $A_{max\_min}=\int_{t=0}^{t_{O\_min}}O_{2}\left(t\right)dt$; area under the oxygen consumption curve and between **t**_O_90_ and **t**_O_10_ (hPa·h) as $A_{max\_min}=\int_{O\_90}^{O\_10}O_{2}\left(t\right)dt$; time when 10% of oxygen remains to be consumed **t**_O_10_ (h); time variation between **t**_O_90_ and **t**_O_10_ (h) as ∆**t**_O_90_10_ = **t**_O_10_ − **t**_O_90_; time when the area under the kinetic curve is half the total area under the curve (h) as *t_A_*_50_ so that $\int_{t=0}^{t_{A_{50}}}O_{2}\left(t\right)dt=\frac{1}{2}\cdot A_{max\_min}$; and maximum value of the oxygen consumption/rate curve (hPa/h) as $R_{max}=max\left\{-\frac{\partial \;O_{2}\left(t\right)}{\partial t}\right\}$*_._*

### 2.4. Analyses

#### 2.4.1. Color Parameters and Total Polyphenol Index

Visible spectra were obtained from all samples using a PerkinElmer’s LAMBDA 25 UV/vis Spectrophotometer (Waltham, MA, USA) interfaced to a computer. Color analysis was performed on all samples at the beginning and end of the consumption kinetics measurement by measuring at 420, 520 and 620 nm, color intensity as the sum of these absorbances as defined by Glories [28]. The same total phenolic index (TPI) was analyzed by the Ribereau–Gayon [29] method. All parameters were measured in duplicate making a total of 96 analyses (3 GEw × 16 conditions × 2 analyses).

#### 2.4.2. Antioxidant Capacity

Antioxidant capacity was measured by ABTS and DPPH. For ABTS, the Re et al. [30] method was followed with some modifications. Briefly, an ABTS (Sigma Aldrich, Steinheim, Germany) was dissolved in water to a 7 mM concentration. Then, ABTS radical cation (ABTS+) was produced by reacting with 2.45 mM potassium persulfate (Sigma Aldrich, Steinheim, Germany) in dark and room temperature for 12–16 h. Then, it was diluted 1:100 with ethanol (AGR ACS ISO, Labbox Labware, Barcelona, Spain) (absorbance at 734 nm was 0.70 ± 0.02). For determination, 50 µL of the diluted sample was mixed with 1.95 mL of ABTS+ and incubated at 35 ± 0.2 °C for 50 min. For DPPH analysis, the Brand-Williams et al. (1995) [31] method was considered with some modifications. Briefly, a DPPH (Sigma Aldrich, Steinheim, Germany) solution was dissolved in ethanol to a 6 × 10^−5^ M (absorbance at 515 nm was 0.50–0.70). For determination, 50 µL of the diluted sample was mixed with 1.95 mL DPPH and incubated at 25 ± 0.2 °C for 60 min.

For both methods, the dilution was made (1:50). Milli-Q water was used for the blank. Trolox (Sigma Aldrich, Steinheim, Germany) was used as standard, and the corresponding calibration curve constructed for each trial was performed with five points (from 0.05 to 1 mM) (R2: 0.98–0.99). The samples, blank and Trolox were analyzed in duplicate.

#### 2.4.3. Individual Anthocyanin Analysis

The analysis of the five major anthocyanins in wines, delphinidin-3-O-glucoside (Df-3-Gl), cyanidin-3-O-glucoside (Cn-3-Gl), petunidin-3-O-glucoside (Pt-3-Gl), peonidin-3-O-glucoside (Pn-3-Gl) and malvidin-3-O-glucoside (Mv-3-Gl) was performed using the method described by del Álamo Sanza et al. [32]. Chromatographic separation was performed on a Fortis C18 column (with a particle size of 5 μm, a length of 250 mm and a diameter of 4.6 mm) (Sugelabor, Spain). Anthocyanins were eluted using a 0.8 mL/min gradient flow rate of solvents A, B and C, with a column temperature of 30 °C. The volume of sample injected was 40 µL and with quantification at 528 nm as it was the predominant one. The quantitative analysis was performed using the external standard method based on malvidin-3-O-glucoside (Mv-3-Gl). The anthocyanin analysis was carried out in duplicate.

## 3. Results and Discussion

### 3.1. Optimization of Reconstitution Conditions

**Figure 1:**
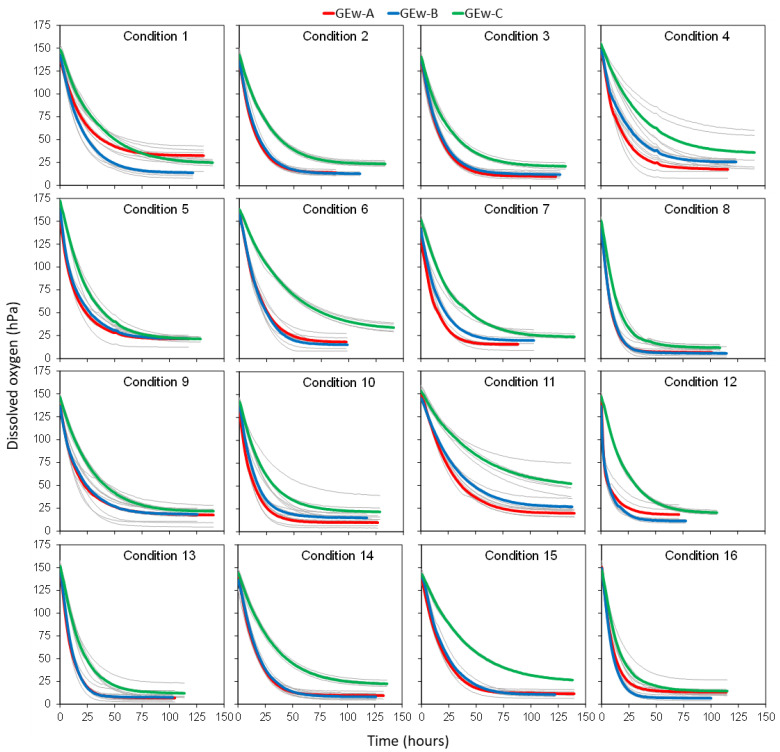
Each one of the 16 graphs represents the oxygen kinetic of a different experimental condition (Table 1). On each graph, the average oxygen kinetics Gew-A, Gew-B and Gew-C were represented in red, blue, and green, respectively, while the 5 replicates of each GEw are shown in gray.

The different reconstitution conditions were applied to the three GEws studied, obtaining the corresponding GEw and their oxygen consumption kinetics. Figure 1 presents, for each of the 16 reconstitution conditions proposed in the Taguchi orthogonal array experiment design, the 15 kinetics curves (five replicas for each GEw) in grey, together with the mean kinetic curve for each GEw in red, blue, and green. The analysis of the consumption kinetics allowed the extraction and calculation of different parameters [15] that defined the oxygen consumption kinetics. These parameters, calculated for each GEw in each condition tested, are shown in Table 2.

The reconstitution conditions of GEws had a decisive influence on the kinetics of oxygen consumption (Figure 1). It was observed that GEws under saturation conditions assumed 172.5 and 131.1 hPa of dissolved oxygen (*O_max_*), as described by del Alamo-Sanza et al. [15] for red wines (154 to 130 hPa). GEw-C reconstituted with condition 5 admitted the most hPa of oxygen, while GEw-A reconstituted with condition 10 was at the opposite extreme. It is interesting to note that the time needed for the wine to consume the dissolved oxygen (*t__min_*) varied from 60.8 to 139.3 h, with a difference of 78.5 h between the fastest GEw-B condition 12 and the slowest GEw-C with condition 6. Table 2 shows how condition 11 is the one that led to a slower consumption of up to 10% oxygen in every GEw (A, B and C), with a longer time t_0_10_ and ∆**t**_O_90_10_ resulting in a higher A_90_10_. Regarding the capacity to consume the available oxygen, it was found that Gews consumed between 96.1% and 66.2% of all dissolved oxygen, leaving from 5.6 hPa to 51.75 hPa unconsumed. The Gew that consumed the greatest amount of oxygen with respect to the initial oxygen was Gew-B in condition 8. Gew-A and Gew-C consumed the greatest amount of oxygen in conditions 8 and 13, very similar consumptions between the two conditions, with an oxygen consumption in the condition 8 with respect to their initial situation of 95.3% and 92.2%, respectively.

Given that the aim of this work was to determine the reconstitution condition that allowed the greatest differentiation between the GEws under study based on the greatest differentiation between their oxygen consumption kinetics, an ANOVA was performed to compare the GEws in pairs on the basis of the parameters obtained in each of the oxygen consumption kinetics of the 16 conditions tested. Thus, it was analyzed whether in condition 1 there were statistically significant differences for the parameters of the consumption kinetics that defined GEw-A and GEw-B, obtaining the corresponding *p*-level_A–B_. We also inquired whether there were differences between GEw-A and GEw-C, obtaining the corresponding *p*-level_A–C_; and finally, between GEw-B and GEw-C, obtaining the corresponding *p*-level_B–C_. The worst significance, that is, the highest *p*-level among the three obtained (*p*-level_A–B_*, p*-level_A–C_ *or p*-level_B–C_) was selected as the aptitude indicator for each of the parameters. Thus, for example for the ∆O_max_min_, the aptitude in condition 1 is 0.0168 (Table 3). This indicated that when one parameter of the kinetics has a significant aptitude indicator, that parameter differed significantly in the kinetics of the three GEw compared two by two. The same was performed for the other 10 parameters of the kinetics developed in condition 1 and for the 11 parameters of the kinetics developed in the other 15 conditions studied (Table 3). The response optimization analysis indicated that the ideal reconstitution condition for the differentiation of the three GEw would be condition 11, since it showed statistically significant differences in the 11 parameters studied. Thus, the reconstitution conditions were set at 12% (*v*/*v*) alcoholic strength; pH: 3.3; 1 mg/L Fe^2+^; 0.1 mg/L Cu^2+^; 1 mg/L Mn^2+^ and 10 mg/L acetaldehyde.

### 3.2. Effect of Reconstitution Parameters on Oxygen Consumption Kinetics

**Figure 2:**
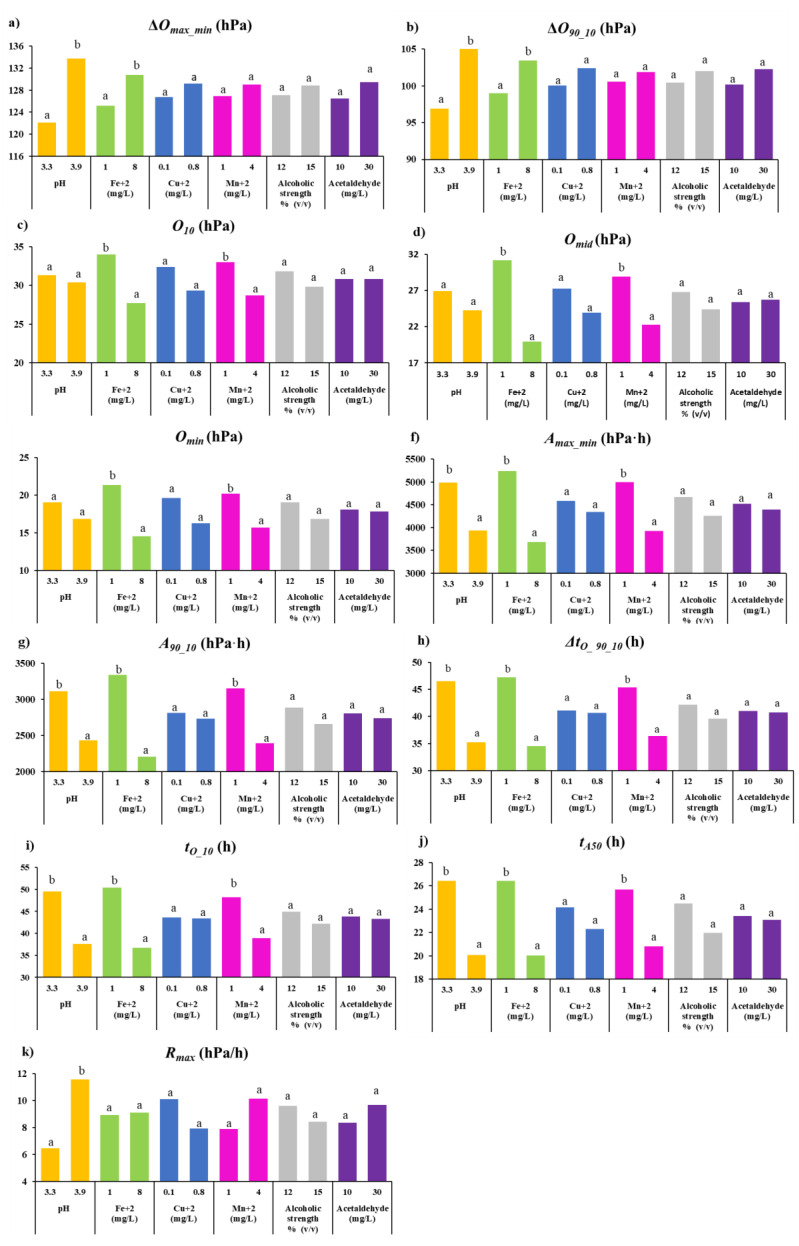
Graphical representation of average value of each parameter of the oxygen consumption kinetics for each concentration level of the different input parameters used for reconstitution. For the same color, different letters indicate significant differences between different values or concentrations of reconstitution input parameters according to Fisher’s LSD test (*p* < 0.05). (**a**) **∆*O_max_min_***: total oxygen consumed (hPa); (**b**) **∆*O_90_10_***: variation between oxygen 90% and oxygen 10% (hPa); (**c**) **O_10_**: oxygen value that represents 10% of the range between the maximum and minimum values (hPa); (**d**) ***O_mid_***: oxygen at half consumption time (hPa); (**e**) ***O_min_***: minimum/final oxygen value (hPa); (**f**) ***A_max_min_***: area under the oxygen consumption curve (hPa·h); (**g**) ***A_90_10_***: area under the oxygen consumption curve and between ***t**_O_90_* and *****t**_O_10_*** (hPa·h); *****t**_O_10_***: time when **O_10_** is reached; (**h**) **∆**t**_O_90_10_**: time variation between *****t**_O_90_*** and (**i**) *****t**_O_10_***; (**j**) ***t_A50_***: time when the area under the kinetic curve is half the total area under the curve (h); (**k**) ***R_max_***: maximum value of the oxygen consumption/rate curve (hPa/h).

Table 4 shows the Pearson correlation coefficients between the concentration of the different reconstitution compounds and the value of the 11 parameters for the consumption kinetics of the GEw studied. Figure 2 shows the average value of each parameter of the consumption kinetics for each level of the different input parameters used for reconstitution.

The oxygen-consuming capacity defined by ∆*O_max−min_* and ∆*O_90_10_* in GEws was positively correlated with pH and Fe^2+^. The largest differences were found due to pH. These compounds were also positively correlated with the amount of oxygen that GEw consumed (between 90% and 10% of the total available) (∆*O*_90 10_). Thus, GEws with pH 3.3 consumed on average 122.2 hPa (∆*O_max−min_*) and its value of ∆O_90_10_ was 96.7 hPa, while GEw with pH 3.9 showed higher levels of ∆*O_max−min_*, and ∆O_90_10_ with values of 133.8 hPa and 105.6 hPa, respectively (Figure 2a,b). Oxygen remaining when 90% of initial dissolved oxygen (*O_10_*) had been consumed (Figure 2c) was statistically significantly negatively correlated with iron and manganese (Table 4). The level of dissolved oxygen available just after half of the time required to reach the minimum oxygen (*O_mid_*) was lower when Fe^2+^ and Mn^2+^ were used at higher concentrations (Figure 2d). In the case of the oxygen left unconsumed, the residual (*O_min_*) was significantly lower when the higher Fe^2+^ and Mn^2+^ content was added (Figure 2e)—32% lower in GEw with 8 mg/L Fe^2+^ and 22% lower in GEw with 4 mg/L Mn^2+^. Therefore, the *O_min_* value was negatively and significantly correlated with higher iron and manganese content (Table 4). These consumption kinetics parameters (*O_min,_ O_mid_* and *O_10_*) demonstrated that GEws reconstituted with higher Fe^2+^ and Mn^2+^ content had the capacity to consume more oxygen. This confirms that iron and manganese are catalysts of oxygen consumption by wines, as previously indicated by several authors [25,26,33]. GEws reconstituted at pH 3.9 presented lower mean values for these three parameters, 11% in *O_min_*, 10% in O_mid_ and 3% in O_10_, but without any statistically significant differences in *O_mid_* or *O_min_*, despite having the highest ∆*O_max_min_.*

The kinetics parameters related to area, and which contributed to greater differentiation were *A_max_min_* and A_90_10_. Figure 2f shows how *A_max_min_* was significantly lower when the higher pH, Fe^2+^ and Mn^2+^ were used. The highest *A_max_min_* was obtained when 1 mg/L Fe^2+^ was used to reconstitute the GEw, being 5239.7 hPa·h, followed by 4999.4 hPa·h with 1 mg/L Mn^2+^ and 4988.0 hPa·h at pH 3.3 (Figure 2f). The values found for GEw were between those described for red wines by del Alamo-Sanza et al. [15] (1762–12341 hPa·h. The differences for *A_max_min_* based on Fe^2+^ reconstitution with 1 or 8 mg/L was 30%, with a negative correlation of -0.3509. The differences between *A_max_min_* and the different pH and Mn^2+^ levels were 21% and 22%, respectively (Figure 2f), presenting negative correlations of −0.2375 and −0.2426 (Table 4). A_90_10_ shows that avidity was higher even when the extremes of the curve were not considered, increasing the differences between the different levels used for the reconstitution of Fe^2+^, pH and Mn^2+^, these being 34%, 22% and 24%, respectively (Figure 2g).

The different concentrations of Fe^2+^ and Mn^2+^ and the pH used for the reconstitution of GEw value were those that showed a significant influence on the time-related parameters of oxygen consumption kinetics. ∆**t**_O_90_10_ was statistically significantly lower when GEw were reconstituted with higher pH, Fe^2+^ and Mn^2+^ (Figure 2h) with a negative correlation (Table 4). Therefore, GEw with pH 3.9 took 11 h less than pH 3.3, 13 h less with 8 mg/L than with 1 mg/L of Fe^2+^ and 9 h less with 4 mg/L than 1 mg/L of Mn^2+^. Figure 2i shows that **t**_O_10_ was faster at pH 3.9 (12 h less), at 8 mg/L Fe^2+^ (13.7 h less) and at 4 mg/L Mn^2+^ (9 h less) than the GEw with pH 3.3, 1 mg/l of Fe^2+^ and 1 mg/l Mn^2+^, respectively. t_A50_ was on average 24% lower, the higher the pH and Fe^2+^ content used, and 19% lower on average when the higher Mn^2+^ was added (Figure 2j), presenting a negative and statistically significant correlation (Table 4). However, *R_max_* only showed statistically significant differences based on pH (Figure 2k)—11.6 hPa/h at pH 3.9 and almost half (6.5 hPa/h) at pH 3.3, showing a significant correlation (Table 4).

*R_max_* parameter has been described as higher when the metal content is higher. However, as can be seen in Figure 2k, there were no statistically significant differences for this parameter for any of the metals studied: even the trend observed for Cu^2+^ is the reverse. The study of commercial wines indicated that *O_mid_* and ∆**t**_O_90_10_ were the parameters that showed the greatest ability to differentiate wines [15]. The two input parameters with a significant effect were Fe^2+^ and Mn^2+^ (Figure 2, Table 4). Figure 2 shows that the level used for the remaining input parameters of Cu^2+^, alcohol strength and acetaldehyde reconstitution did not show any statistically significant differences in any of the kinetic parameters studied, and no correlations were found (Table 4).

The difference in pH between the maximum and minimum value used was more widely involved in the consumption capacity (∆*O_max_min_,* ∆*O_90_10_* and *R_max_*), affecting all the parameters related to the time. It has been observed that an increase in pH accelerates oxygen consumption in wines [10,22]. Singleton [22] indicated that the autooxidation of a wine should be nine times faster at pH 4.0 than at pH 3.0. Ferreira et al. [10] found a significant and positive correlation between mean oxygen consumption rates in a red wine and pH, highlighting the importance of pH in oxidation reactions. However, when models are made to study oxygen consumption in wines, this parameter is not usually considered. This work shows that it is essential to take pH into account when reconstituting GEw, pH 3.3 being the value that allowed a better differentiation, always considering that the effect was not due to a single factor but rather to a set. In our case, the rate of consumption at pH 3.9 was almost twice as fast as at pH 3.3.

Metals are important factors influencing oxygen consumption in wine, as they are catalysts of oxidative reaction [25,26,33]. del Alamo-Sanza et al. [15] found no significant correlations between *O_min_* and the initial iron or copper content of the wines. The iron concentrations in this work ranged from 1.10 to 2.68 mg/L and copper from 0.09 to 0.15 mg/L [15]. The major differences in the present work between the lowest and highest Fe^2+^ level (7 mg/L) could be responsible for the differences for *O_min_*. That difference in Fe^2+^ concentration in PAwF caused them to consume 6.8 hPa more when reconstituted with the higher level (Figure 2e), with a negative correlation of −0.3238 with *p* < 0.005 between *O_min_* and Fe^2+^ (Table 4). However, the difference between 0.1 and 0.8 mg/L Cu^2+^ was not significant, obtaining very similar values of O_min_, with 19.6 hPa and 16.3 hPa, respectively. Overall, the values for this parameter shown by GEw were like those found by del Alamo-Sanza [15]. Rousseva et al. [34] found correlations between Fe^2+^ and Cu^2+^ concentrations and wine oxygen consumption, with total copper correlating more closely with oxygen consumption compared to total iron. The iron and copper contents of the wines studied by these authors ranged from 0.31 to 11.3 mg/L for iron and from 0.69 to 6.52 mg/L for copper, indicating differences between the different concentrations of the metals much higher than those used in the reconstitution of GEw, especially for copper (Table 1). Nevares et al. [13] found correlations between copper and iron content, especially iron, and the three characteristics used to describe the rate of oxygen consumption kinetics of the wines were even higher than those found with total polyphenols (TP). For GEw, *R_max_* were higher when Fe^2+^ was higher, although this was not statistically significant, and the reverse was the case for the copper content, showing negative correlations. t_A50_, which reports the time in which the area under the consumption kinetics curve is half of the total area under the curve, was previously described as OCRI by Nevares et al. [13]. These authors did not observe any correlation between the chemical content of white wines and OCRI, but they did in red wines, where it was observed that iron and total acidity presented a negative correlation. These results coincided with those observed in the present study, where Fe^2+^ and pH used to reconstitute GEw were negatively correlated with t_A50_ (Table 4). Ferreira et al. [10] observed that oxygen consumption rates were independent of iron, finding a positively trending correlation between initial oxygen consumption rate and copper, and between average oxygen consumption rate and magnesium, but no statistically significant differences. Danilewicz [18] observed that copper accelerated Fe^2+^ oxidation and, therefore, could greatly accelerate wine oxidation. Kontoudakis and Clark [35] studied the relationship between the two main forms of copper, sulfur-bound or not, in wine and their impact on oxygen consumption rates and observed that wines in which ascorbic acid had not been added copper had little effect on oxygen consumption rates. The addition of 5 g/hL of potassium metabisulfite to the GEw and the non-addition of ascorbic acid were probably what accounted for Cu^2+^ not affecting oxygen consumption or the consumption rate, since its binding to sulfur did not allow its involvement in oxygen consumption.

Marrufo-Curtido et al. [36] stated that manganese plays an important role in the pre-initial and middle oxygen consumption stages. Danilewicz [18] proposed that manganese, which theoretically only has a stable redox state in acid medium as Mn^2+^, reacts with a Fe^3+^ (III)-superoxide complex, generating the strong oxidant Mn^3+^, observing that small amounts of this oxidant would suffice to accelerate the oxidation of the different polyphenols in wine. This work showed the enhancing effect of manganese on oxygen consumption in white wines, which had also been observed previously by Ferreira et al. [10]. In this paper, a correlation between manganese and most of the parameters of the consumption kinetics of GEw from red grapes was observed, reflecting the importance of this parameter on oxygen consumption as had already been observed in white wines, since the effect of this metal on oxygen consumption in red wines has not been found in the literature. Thus, the difference of 3 mg/L of Mn^2+^ between the maximum and minimum manganese used during reconstitution resulted in an increase in the parameters related to the area *A_max_min_* and *A_90_10_*, together with lower oxygen values of *O_min_*, *O_mid_*, and *O_10_,* as well as shorter times spent in consuming different amounts of oxygen, ***t**_O_10_*, ∆**t**_O_90_10_ and t*_A50_*. However, it did not significantly affect the rate of oxygen consumption (*R_max_*) (Figure 2, Table 4).

Different works reported that the initial acetaldehyde in wines is correlated with oxygen consumption. Carrascón et al. [7] observed a negative correlation between the initial acetaldehyde content of wines and the initial oxygen consumption rate. This study indicated that this negative role of acetaldehyde on the oxygen consumption rate of wine could be related to its ability to interact with SO_2_. Marrufo-Curtido et al. [36] established six models for the measurement of oxygen consumption and in all of them acetaldehyde played an important role, being negatively correlated. However, reconstitution of GEw with 10 or 30 mg/L acetaldehyde did not affect any of the parameters of oxygen consumption kinetics under the conditions studied.

Nevares et al. [13] indicated that it is not enough to know the individual chemical properties to define the oxygen consumption rate of wines, but that it is necessary to evaluate the chemical properties of the wines. Therefore, the great variability of results obtained between the correlations of parameters measuring oxygen consumption and each chemical component of the wines observed in the literature highlight the importance of considering all the components, as well as their concentrations.

### 3.3. Effect of the Initial Phenolic Composition of GEws on the Modification of Oxygen Consumption Kinetics

It is widely known that phenolic compounds influence the ability of wine to consume oxygen [10,36,37]. Therefore, the effect of the initial chemical parameters of GEw (anthocyanin content, antioxidant capacity, total phenol concentration, absorbance at different wavelengths and color intensity) on the parameters defining the oxygen consumption kinetics is described below. Table 5 shows the effect of the initial chemical parameters of GEw on the oxygen consumption kinetics parameters for each of the three GEws reconstituted with the conditions under which the greatest differentiation (condition 11). GEw-A and GEw-B had a similar initial composition, resulting in fewer differences in the parameters studied.

GEw-A presented higher contents of Df-3-Gl, Pt-3-Gl and Mv-3-Gl, 21%; 17% and 11% more than GEw-B; and 17%, 93% and 60% more than GEw-C. IPT was also higher in GEw-A—7% more than GEw-B and 51% more than GEw-C. Many authors have highlighted the existence of a positive correlation between monomer anthocyanins and IPT of wines, and antioxidant capacity [38,39,40,41,42]. Therefore, as would be expected, GEw-A had a higher antioxidant capacity (Table 5). The ABTS and DPPH values for GEw-A were 17% and 9% higher than GEw-B, respectively, and 48% and 62% higher than GEw-C. In addition, GEw-A had the highest color intensity, presenting 1.6 points more than GEw-B and 6.3 more than GEw-C, with higher absorbances at all wavelengths.

GEw-C presented statistically higher values of *A_max_min_* and *A*_90*_*10_, with greater differences for the latter parameter, with 53% and 43% more than GEw-A and GEw-B, respectively. Higher values of *A_max_min_* are related to lower oxygen consumption ∆*O_max_min_*, GEw-C thus having the lowest value of this parameter with 101.52 hPa. This lower avidity presented by GEw C meant that the residual dissolved oxygen was not able to be consumed by the wine (*O_min_*) was 51.8 hPa, compared to 26.5 hPa for GEw-B and 19.44 hPa for GEw-A. Since the presence of additives was the same, both for sulfur dioxide added during the time of obtaining GEws from grapes, as well as for the metals considered in this study (those respective to condition 11), these higher oxygen consumptions in GEw-A and B and the higher final oxygen contents of GEw-C are related to the initial chemical composition of GEw-C. This is because the phenolic composition of wine is related to its oxygen avidity, and wines with a higher content of phenolic compounds tend to cause higher oxygen consumption [10,15,43]. This lower oxygen consumption avidity of GEw-C was particularly observed through the *O_mid_*. At this time, a difference between GEw-C and GEw-B of 31.8 hPa was observed, which was slightly lower for *O_10_*, with 23.5 hPa and of 25.2 hPa at the end of the consumption process (*O_min_*). The differences were larger when compared to the GEw-A data, largely due to the greater differences in initial chemical composition—42.8, 29.5 and 32.3 hPa in *O_mid_*, *O_10_*, and *O_min_*, respectively. Furthermore, comparison of the three GEws in condition 11 showed that there were statistically significant differences between the Gew with the greatest phenolic difference (Gew-A and Gew-C) in all parameters of consumption kinetics related to area (*A_max_min_* and *A_90_10_*) and to time at the different moments (***t**_O_10_*), ∆**t**_O_90_10_ and *t_A50_*), which were higher for the Gew with lower phenolic content, i.e., in Gew-C. Thus, the latter Gew-C presented the highest area values and needed 83.4 h to consume 80.9 hPa while Gew-A needed 52.20 h to consume 103.6 hPa and Gew-B needed 59.9 h to consume 94.26 hPa. Therefore, the parameter referring to the rate of consumption (*R_max_*) was statistically lower in Gew-C and thus was the slowest kinetic. This showed that not only a higher phenolic content meant a higher oxygen consumption, but also a higher avidity which was demonstrated by a faster consumption. Thus, the values of this parameter were 23.5% faster in Gew-A than in Gew-B and 31.7% faster when compared to Gew-C.

## 4. Conclusions

A method has been developed to determine the oxygen consumption of wine that could be obtained from grape extracts (with phenols and aromas). The work has shown that reconstitution of Ges could be carried out under conditions that ensure the greatest differentiation on oxygen consumption kinetics among Ges. The reconstitution levels of the parameters considered (pH, copper, iron, manganese, alcoholic strength, and acetaldehyde) that cause the maximum differentiation between the different grape extracts (GEs) studied were pH: 3.3; Fe^2+^: 1 mg/L; Cu^2+^: 0.1 mg/L; Mn^2+^: 1 mg/L, alcoholic strength 12% (*v*/*v*) and acetaldehyde 10 mg/L. The reconstitution conditions had a decisive influence on the kinetics of oxygen consumption. The parameters that had the greatest effect on the consumption kinetics (∆*O_max_min_* and ∆*O_90_10_*) of GEws were pH, Fe^2+^ and Mn^2+^, as evidenced by significant correlations with the 11 parameters extracted from the oxygen consumption kinetics. However, Fe^2+^ and Mn^2+^ behaved as catalysts since higher reconstitution contents of the GEw with these presented the ability to consume more oxygen. The highest avidity and shortest time for oxygen consumption was shown by the GEws with high reconstitution levels of pH, Fe^2+^ and Mn^2+^. A lower phenolic content means a lower avidity and a longer consumption time, which is demonstrated by a slower consumption rate.

The results show that with the optimal reconstitution of GEs to know their consumption kinetics, we have an interesting tool for the classification and evaluation of grapes according to their oxidability potential, which would allow for predicting the sensitivity of the must/wine to oxygen during its elaboration.

## Figures and Tables

**Table 1:** The experimental conditions in L16 (2^15^) Taguchi orthogonal array for the study the oxygen consumption potential of wines.

Condition	pH	Fe^2+^ (mg/L)	Cu^2+^ (mg/L)	Mn^2+^ (mg/L)	Alcoholic Strength (*v*/*v*)	Acetaldehyde (mg/L)
1	3.3	1	0.1	4	15	30
2	3.3	8	0.1	1	15	30
3	3.3	8	0.1	4	12	10
4	3.9	1	0.8	1	12	10
5	3.9	8	0.1	1	12	30
6	3.9	1	0.8	4	15	30
7	3.9	1	0.1	1	15	10
8	3.9	8	0.8	4	12	10
9	3.3	8	0.8	1	12	30
10	3.3	8	0.8	4	15	10
11	3.3	1	0.1	1	12	10
12	3.9	1	0.1	4	12	30
13	3.9	8	0.8	1	15	30
14	3.3	1	0.8	4	12	30
15	3.3	1	0.8	1	15	10
16	3.9	8	0.1	4	15	10

**Table 2:** Mean and standard deviation of each consumption kinetic parameter in the different experimental conditions for each GEw.

Condition	$∆O_{max\_min}$	$∆O_{90\_10}$	$O_{10}$	$O_{mid}$	$O_{min}$	$A_{max\_min}$	$A_{90\_10}$	$∆t_{O\_90\_10}$	$t_{O\_10}$	$t_{A_{50}}$	$R_{max}$
	**GEw-A**										
1	106.9 ± 9.7	85.0 ± 7.6	43.3 ± 9.8	40.5 ± 11.8	32.5 ± 10.7	5916.7 ± 1803.9	3413.5 ± 986.3	46.5 ± 9.9	48.8 ± 10.0	33.2 ± 10.5	5.6 ± 0.8
2	122.9 ± 7.9	96.9 ± 6.0	26.7 ± 2.3	20.5 ± 3.6	14.2 ± 2.7	2859.4 ± 320.7	1774.3 ± 116.8	29 ± 1.1	31.4 ± 1.0	16.1 ± 2.3	8.3 ± 1.2
3	127.3 ± 3.9	100.7 ± 3.0	22.9 ± 2.5	13.7 ± 3.0	10.1 ± 2.4	3237.9 ± 380.7	1920.5 ± 148.0	33.6 ± 1.0	36.3 ± 1.5	17.8 ± 1.8	8.3 ± 0.6
4	128.5 ± 6.9	101.6 ± 5.4	30.9 ± 5.8	27.8 ± 7.0	17.9 ± 6.4	3848.8 ± 1191.3	2368.3 ± 595.2	36.8 ± 10.1	39.5 ± 10.2	21.0 ± 9.1	8.1 ± 0.6
5	129.7 ± 4.5	103.0 ± 3.6	34.4 ± 4.7	28.4 ± 7.4	21.3 ± 5.2	4138.0 ± 735.4	2411.3 ± 437.9	36.3 ± 6.6	38.2 ± 6.5	23.8 ± 5.9	10.0 ± 0.5
6	141.0 ± 4.9	112.1 ± 4.0	32.3 ± 5.5	28.4 ± 6.2	18.1 ± 5.9	3992.6 ± 645.3	2554.0 ± 461.5	35.5 ± 6.9	38.1 ± 7.1	18.4 ± 4.0	7.7 ± 0.9
7	127.0 ± 4.6	99.5 ± 3.1	28.3 ± 1.3	22.5 ± 2.8	15.5 ± 1.5	2534.0 ± 45.5	1667.5 ± 40.9	26.7 ± 0.6	27.5 ± 0.6	14.6 ± 0.8	19.7 ± 1.0
8	135.1 ± 2.8	106.1 ± 2.2	20.3 ± 2.0	8.4 ± 2.5	6.6 ± 1.9	1823.5 ± 232.4	1031.6 ± 66.7	17.8 ± 1.3	19.5 ± 1.4	9.5 ± 1.6	12.6 ± 1.2
9	119.9 ± 9.3	95.0 ± 7.6	29.6 ± 9.4	22.7 ± 13.8	17.5 ± 10.3	4384.3 ± 1536.1	2574.7 ± 976.2	42.5 ± 13.4	44.5 ± 13.2	27.9 ± 11.5	7.6 ± 0.6
10	121.2 ± 4.8	95.8 ± 3.2	22.1 ± 5.0	14.0 ± 7.1	10.0 ± 5.4	2432.4 ± 565.9	1482.2 ± 259.8	27.3 ± 3.9	28.8 ± 3.7	15.3 ± 5.0	9.4 ± 0.7
11	130.0 ± 1.7	103.6 ± 1.1	32.5 ± 4.0	28.7 ± 4.4	19.4 ± 3.9	5767.0 ± 431.6	3583.8 ± 254.1	52.2 ± 1.4	56.3 ± 1.6	28.0 ± 1.8	5.6 ± 0.7
12	128.5 ± 6.1	97.6 ± 4.6	31.1 ± 6.9	22.3 ± 9.7	18.1 ± 7.5	1967.5 ± 580.6	944.8 ± 358.8	17.4 ± 5.0	18.1 ± 5.0	16.3 ± 3.6	33.0 ± 2.6
13	141.2 ± 2.7	111.7 ± 2.0	21.1 ± 4.6	10.3 ± 5.3	6.9 ± 4.8	2034.7 ± 381.5	1295.5 ± 115.6	21.8 ± 1.6	23.4 ± 1.6	10.1 ± 2.7	12.5 ± 1.0
14	129.4 ± 3.9	102.7 ± 3.4	22.5 ± 3.0	11.8 ± 3.6	9.4 ± 3.3	3181.6 ± 312.9	1893.8 ± 72.6	31.9 ± 0.3	34.5 ± 0.7	17.1 ± 3.0	8.6 ± 0.5
15	126.7 ± 1.9	100.1 ± 1.2	24.3 ± 3.8	15.5 ± 3.8	11.5 ± 3.8	3650.8 ± 475.9	2235.2 ± 157.1	37.2 ± 1.3	40.0 ± 2.0	19.7 ± 2.7	6.7 ± 0.2
16	137.3 ± 6.4	107.8 ± 5.6	27.2 ± 2.5	15.8 ± 4.3	13.3 ± 2.8	2660.0 ± 310.9	1346.0 ± 197.6	21.1 ± 3.6	22.7 ± 3.7	15.7 ± 2.5	12. ± 1.7
	**GEw-B**										
1	128.5 ± 7.6	101.6 ± 6.2	26.7 ± 6.0	19.8 ± 7.3	13.8 ± 6.5	4202.0 ± 751.7	2587.2 ± 367.5	42.4 ± 2.6	45.3 ± 2.5	22.6 ± 5.0	6.4 ± 0.4
2	122.8 ± 8.5	97.0 ± 6.4	25.5 ± 2.5	17.6 ± 2.9	13.1 ± 1.8	3237.8 ± 289.0	1938.7 ± 203.3	33.0 ± 0.7	35.2 ± 0.7	18.5 ± 0.6	7.9 ± 1.2
3	126.9 ± 3.7	100.7 ± 3.1	25.0 ± 3.1	15.6 ± 2.2	12.2 ± 3.1	3635.3 ± 363.5	2055.9 ± 90.2	35.1 ± 1.9	37.7 ± 2.0	21.0 ± 3.7	7.7 ± 0.5
4	122.2 ± 2.0	96.8 ± 1.4	38.3 ± 3.4	36.0 ± 5.6	26.0 ± 3.5	5348.6 ± 382.1	3218.3 ± 319.1	46.4 ± 4.1	49.1 ± 4.1	29.1 ± 2.5	7.2 ± 0.8
5	141.7 ± 1.4	112.0 ± 1.9	36.1 ± 1.5	29.8 ± 2.4	21.8 ± 1.5	4579.4 ± 363.8	2740.5 ± 309.2	39.4 ± 3.4	41.1 ± 3.3	25.2 ± 1.5	12.3 ± 1.5
6	142.7 ± 6.8	113.4 ± 5.8	29.5 ± 4.4	28.5 ± 4.5	15.0 ± 5.0	3755.0 ± 618.2	2428.0 ± 208.1	34.2 ± 3.0	37.1 ± 3.1	16.7 ± 3.8	8.1 ± 1.0
7	122.2 ± 11.8	97.0 ± 9.2	32. ± 7.2	27.4 ± 9.3	19.7 ± 8.3	3710.0 ± 1089.5	2331.0 ± 594.5	35.1 ± 6.9	36.9 ± 7.2	20.2 ± 6.8	9.3 ± 2.5
8	136.7 ± 1.4	108.1 ± 1.5	19.4 ± 4.3	7.2 ± 5.1	5.6 ± 4.6	1833.2 ± 439.1	1088.5 ± 96.1	19.0 ± 0.8	20.5 ± 0.8	9.8 ± 3.5	13.0 ± 0.9
9	118.2 ± 6.0	93.7 ± 4.4	30.1 ± 5.4	26.4 ± 4.7	18.2 ± 6.0	4236.8 ± 924.0	2579.4 ± 347.8	43.2 ± 4.9	45.1 ± 4.9	26.8 ± 7.3	7.3 ± 1.0
10	125.3 ± 4.2	99.4 ± 3.1	27.9 ± 4.8	18.0 ± 6.0	15.2 ± 5.0	3377.2 ± 773.0	1811.0 ± 457.5	29.1 ± 7.5	30.9 ± 7.3	20.7 ± 6.3	8.8 ± 0.4
11	118.8 ± 6.3	94.3 ± 5.3	38.5 ± 5.4	37.6 ± 4.6	26.5 ± 6.0	7012.7 ± 692.0	4322.6 ± 257.8	59.9 ± 3.4	64.3 ± 3.3	35.3 ± 4.2	4.3 ± 0.3
12	125.4 ± 0.6	95.1 ± 2.6	23.8 ± 1.7	15.2 ± 2.0	11.1 ± 1.8	1567.5 ± 132.6	903.1 ± 65.6	18.9 ± 1.0	19.6 ± 0.9	12.2 ± 1.5	30.7 ± 0.7
13	143.3 ± 3.0	112.7 ± 2.2	22.1 ± 3.7	9.2 ± 4.5	7.4 ± 3.4	2160.6 ± 268.3	1310.3 ± 26.6	20.8 ± 1.6	22.5 ± 1.8	10.3 ± 2.1	12.3 ± 1.7
14	128.4 ± 4.3	101.6 ± 3.3	21.1 ± 3.5	13.4 ± 5.3	8.1 ± 3.3	3110.7 ± 370.9	2005.3 ± 142.6	35.4 ± 0.6	38.5 ± 1.1	15.6 ± 1.3	7.9 ± 0.3
15	132.0 ± 3.9	104.8 ± 3.3	23.8 ± 1.9	17.4 ± 4.5	10.5 ± 2.0	3926.0 ± 305.4	2531.3 ± 271.9	42.8 ± 2.3	46.2 ± 2.6	19.9 ± 1.3	6.9 ± 0.4
16	142.8 ± 4.1	112.6 ± 3.3	21.1 ± 1.7	9.4 ± 3.1	6.6 ± 1.7	1849.2 ± 141.5	1163.5 ± 47.7	19.1 ± 1.5	20.7 ± 1.5	8.7 ± 1.3	13.2 ± 1.1
	**GEw-C**										
1	123.6 ± 0.8	98.2 ± 0.7	37.3 ± 2.7	38.0 ± 2.6	24.9 ± 2.6	7223.6 ± 425.4	4666.3 ± 198.2	65.8 ± 0.9	70.0 ± 1.0	36.3 ± 1.5	4.6 ± 0.4
2	118.9 ± 1.9	94.4 ± 1.7	35.8 ± 2.6	32.2 ± 1.7	23.9 ± 2.4	5943.1 ± 486.5	3595.0 ± 155.9	52.1 ± 0.7	55.3 ± 0.5	31.9 ± 2.8	5.1 ± 0.4
3	119.4 ± 3.6	94.9 ± 3.0	33.3 ± 2.6	29.7 ± 2.1	21.3 ± 2.8	5539.8 ± 504.8	3383.0 ± 235.2	52.0 ± 2.1	55.1 ± 2.1	30.9 ± 2.1	5.3 ± 0.6
4	118.5 ± 18.6	94.3 ± 15.1	48.3 ± 18.0	50.0 ± 19.8	36.3 ± 19.8	8760.3 ± 2318.2	5651.0 ± 1391.1	68.5 ± 7.3	72.5 ± 7.0	40.4 ± 10.4	4.5 ± 0.9
5	151.1 ± 2.4	120.2 ± 1.9	36.6 ± 1.9	31.6 ± 3.3	21.4 ± 2.2	6270.4 ± 586.8	3952.9 ± 412.5	51.1 ± 3.7	54.5 ± 3.6	27.2 ± 2.3	8.1 ± 1.1
6	129.2 ± 3.2	102.9 ± 2.5	46.8 ± 4.1	53.6 ± 3.6	33.7 ± 4.4	9417.2 ± 442.5	6436.1 ± 214.1	77.3 ± 0.9	82.2 ± 1.1	40.8 ± 1.6	4.4 ± 0.4
7	128.0 ± 4.5	101.8 ± 3.5	36.6 ± 2.2	34.8 ± 2.4	23.7 ± 2.3	6664.5 ± 142.5	4184.1 ± 138.4	59.1 ± 1.7	62.9 ± 2.0	33.7 ± 1.1	6.2 ± 1.8
8	138.9 ± 2.0	110.1 ± 1.9	25.8 ± 2.8	15.8 ± 2.6	11.8 ± 2.8	3304.0 ± 257.3	1847.4 ± 158.3	28.8 ± 4.4	31.1 ± 4.6	17.0 ± 1.8	9.6 ± 1.2
9	124.8 ± 2.0	99.2 ± 1.4	34.3 ± 2.0	33.8 ± 5.8	21.7 ± 2.1	6105.4 ± 462.4	3989.0 ± 295.5	57.6 ± 2.8	60.9 ± 2.6	30.9 ± 2.6	5.0 ± 0.8
10	120.6 ± 12.2	95.5 ± 9.5	33.8 ± 10.0	29.6 ± 12.1	21.7 ± 11.2	5313.9 ± 1497.4	3199.2 ± 868.3	47.7 ± 8.0	50.7 ± 7.7	28.8 ± 9.3	5.7 ± 0.5
11	101.5 ± 17.5	80.9 ± 13.9	62.0 ± 11.9	71.4 ± 8.9	51.7 ± 13.6	11,098.7 ± 1092.5	7597.9 ± 390.2	83.4 ± 5.6	88.6 ± 5.5	48.6 ± 4.9	3.0 ± 0.4
12	127.3 ± 1.8	101.1 ± 1.7	32.9 ± 1.9	31.1 ± 2.0	20.1 ± 1.9	4777.9 ± 203.1	3023.2 ± 76.4	44.7 ± 0.5	48.1 ± 0.6	23.9 ± 0.9	6.4 ± 0.4
13	139.6 ± 8.5	111.1 ± 6.8	26.1 ± 5.9	18.6 ± 6.7	12.0 ± 6.6	3934.0 ± 705.4	2495.4 ± 254.0	39.0 ± 1.1	41.9 ± 1.4	18.9 ± 4.6	7.4 ± 0.7
14	121.4 ± 1.6	96.8 ± 1.4	34.7 ± 2.7	34.5 ± 2.9	22.4 ± 2.6	6538.6 ± 390.0	4223.2 ± 170.7	61.5 ± 0.4	65.5 ± 0.6	33.1 ± 1.2	5.0 ± 0.7
15	116.2 ± 0.8	92.5 ± 0.7	38.2 ± 0.6	43.7 ± 1.0	26.5 ± 0.6	7780.9 ± 186.3	5326.3 ± 108.2	74.8 ± 0.7	79.9 ± 1.2	38.2 ± 0.2	4.4 ± 0.4
16	132.3 ± 7.9	104.8 ± 6.2	27.8 ± 6.3	20.3 ± 7.9	14.5 ± 7.0	3484.5 ± 687.9	2018.0 ± 376.3	31.8 ± 4.3	34.2 ± 4.2	18.3 ± 4.4	8.8 ± 1.1

**∆*O_max_min_***: total oxygen consumed (hPa); ***O_min_**:* minimum/final oxygen value (hPa); ***A_max_mi_**_n_:* area under the oxygen consumption curve (hPa·h); ***O_mid_***: oxygen at half consumption time (hPa); **O_10_**: oxygen value that represents 10% of the range between the maximum and minimum values (hPa); **∆O_90_10_**: variation between oxygen 90% and oxygen 10% (hPa); **t_O_10_**: time when O_10_ is reached (h); **∆**t**_O_90_10_**: time variation between **t**_O_90_ and **t**_O_10_ (h); ***A_90_10_***: area under the oxygen consumption curve and between **t_O_90_** and **t_O_10_** (hPa·h); ***R_max_***: maximum value of the oxygen consumption/rate curve (hPa/h); ***t_A_*_50_**: time when the area under the kinetic curve is half the total area under the curve (h).

**Table 3:** Global aptitude indicator for each experimental condition and each parameter.

Condition	$∆O_{max\_min}$	$∆O_{90\_10}$	$O_{10}$	$O_{mid}$	$O_{min}$	$A_{max\_min}$	$A_{90\_10}$	$∆t_{O\_90\_10}$	$t_{O\_10}$	$t_{A_{50}}$	$R_{max}$
1	0.0168 *	0.0221 *	0.0035 ***	0.0028 ***	0.0032 ***	0.8046	0.1330	0.0717	0.0904	0.5908	0.3191
2	0.5275	0.8912	0.0266 *	0.0581	0.0309 *	0.5710	0.7116	0.0300 *	0.0205 *	0.3826	0.0778
3	0.6732	0.4474	0.1921	0.0864	0.2887	0.0011 ***	0.0073 **	0.0250 *	0.0128 *	0.3296	0.1745
4	0.4527	0.5050	0.7612	0.3040	0.9384	0.1271	0.1403	0.6598	0.6710	0.7131	0.1706
5	0.0010 ***	0.0037 ***	0.8449	0.9148	0.6735	0.0634	0.2013	0.0915	0.0926	0.2980	0.1197
6	0.0472 *	0.0603	0.0646	0.4649	0.0390 *	0.5900	0.8845	0.9645	0.9318	0.4409	0.7120
7	0.9062	0.9630	0.0913	0.0896	0.1823	0.0074 **	0.0150 *	0.0174 *	0.0160 *	0.0668	0.1360
8	0.4156	0.4807	0.0444 *	0.0426 *	0.0383 *	0.2776	0.9977	0.5280	0.5851	0.2516	1.0000
9	0.4079	0.3137	0.6012	0.4915	0.3060	0.2559	0.1342	0.3483	0.3055	0.2501	0.6589
10	0.6408	0.7128	0.2639	0.9559	0.3715	0.2559	0.3733	0.6401	0.7298	0.8790	0.0332 *
11	0.0077 **	0.0106 *	0.0243 *	0.0124 *	0.0171 *	0.0129 *	0.0110 *	0.0238 *	0.0270 *	0.0113 *	0.0098 **
12	0.1931	0.7773	0.0519	0.5797	0.2410	0.6553	0.0284 *	0.0338 *	0.0350 *	0.3568	0.0041 ***
13	0.7179	0.7659	0.5826	0.6910	0.6645	0.3672	0.7406	0.1369	0.1443	0.6070	0.4456
14	0.6308	0.4698	0.6697	0.2229	0.6316	0.2778	0.0526	0.0005 ***	0.0007 ***	0.7923	0.2732
15	0.1202	0.1120	0.3121	0.9195	0.2450	0.5953	0.0186 *	0.0105 *	0.0252 *	0.4860	0.5687
16	0.8931	0.8005	0.0497 *	0.5910	0.0150 *	0.1899	0.1192	0.2898	0.2754	0.1945	0.3860

**∆*O_max_min_***: total oxygen consumed (hPa); ***O_min_**:* minimum/final oxygen value (hPa); ***A_max_mi_**_n_:* area under the oxygen consumption curve (hPa·h); ***O_mid_***: oxygen at half consumption time (hPa); ***O*_10_**: oxygen value that represents 10% of the range between the maximum and minimum values (hPa); **∆*O*_90_10_**: variation between oxygen 90% and oxygen 10% (hPa); **t_0_10_**: time when O_10_ is reached (h); ∆**t**_O*_*90_10_: time variation between **t**_O_90_ and **t**_O_10_ (h); ***A_90_10_***: area under the oxygen consumption curve and between **t**_O_90_ and **t**_O_10_ (hPa·h); ***R_max_***: maximum value of the oxygen consumption/rate curve (hPa/h); ***t_A_*_50_**: time when the area under the kinetic curve is half the total area under the curve (h). Significant values of the aptitude indicator are typed in bold according to Fisher’s LSD test: * *aptitude* < 0.05, ** *aptitude* < 0.01 and *** *aptitude* < 0.005.

**Table 4:** Pearson correlation coefficients between the reconstitution compounds concentrations and consumption kinetics parameters of GEws.

	$∆O_{max\_min}$	$∆O_{90\_10}$	$O_{10}$	$O_{mid}$	$O_{min}$	$A_{max\_min}$	$A_{90\_10}$	$∆t_{O\_90\_10}$	$t_{O\_10}$	$t_{A_{50}}$	$R_{max}$
pH	0.5050 ***	0.4794 ***	−0.0488	−0.0937	−0.1028	−0.2375 *	−0.2268 *	−0.3339 ***	−0.3368 ***	−0.3080 ***	0.4427 ***
Fe^2+^ (mg/L)	0.2421 *	0.2817 **	−0.3203 ***	−0.3970 ***	−0.3238 ***	−0.3509 ***	−0.3775 ***	−0.3777 ***	−0.3830 ***	−0.3102 ***	0.0169
Cu^2+^ (mg/L)	0.1091	0.1576	−0.1572	−0.1166	−0.1586	−0.0528	−0.0272	−0.0118	−0.0067	−0.0891	−0.1883
Mn^2+^ (mg/L)	0.0916	0.1139	−0.2192*	−0.2336 *	−0.2148 *	−0.2426 *	−0.2543 *	−0.2671 **	−0.2617 **	−0.2358 *	0.1959
Alcoholic strength (*v*/*v*)	0.0766	0.0676	−0.102	−0.0840	−0.1036	−0.0940	−0.0769	−0.0785	−0.0777	−0.1221	−0.1043
Acetaldehyde (mg/L)	0.1283	0.1597	−0.0004	0.0118	−0.0146	−0.0287	−0.0227	−0.0095	−0.0143	−0.0169	0.1132

∆*O_max_min_*: total oxygen consumed (hPa); *O_min_*: minimum/final oxygen value (hPa); *A_max_min_*: area under the oxygen consumption curve (hPa·h); *O_mid_:* oxygen at half consumption time (hPa); *O*_10_: oxygen value that represents 10% of the range between the maximum and minimum values (hPa); ∆O_90_10_: variation between oxygen 90% and oxygen 10% (hPa); **t**_O_10_: time when O_10_ is reached (h); ∆**t**_O_90_10_: time variation between **t**_O_90_ and **t**_O_10_ (h); *A*_90*_*10_: area under the oxygen consumption curve and between **t**_O_90_ and **t**_O_10_ (hPa·h); *R_max_:* maximum value of the oxygen consumption/rate curve (hPa/h); t_A50_: time when the area under the kinetic curve is half the total area under the curve (h). Significant correlation values are typed in bold according to Pearson test: * *p* < 0.05. ** *p* < 0.01 and *** *p* < 0.005.

**Table 5:** Data of consumption kinetics parameters and initial chemical parameters of each GEw (A, B and C) in condition 11.

	GEw-A	GEw-B	GEw-C
	**Consumption Kinetics Parameters**		
$∆O_{\max\_min}$	130.04 ± 2.44 a	118.75 ± 8.91 a	101.52 ± 24.76 a
$∆O_{90\_10}$	103.58 ± 1.60 a	94.26 ± 7.53 a	80.89 ± 19.68 a
$O_{10}$	32.49 ± 5.67 a	38.48 ± 7.65 a	61.95 ± 16.85 a
$O_{mid}$	28.65 ± 6.27 a	37.07 ± 6.51 a	71.43 ± 12.53 b
$O_{min}$	19.44 ± 5.54 a	26.53 ± 8.55 a	51.75 ± 19.21 a
$A_{\max\_min}$	5767.04 ± 610.34 a	7012.69 ± 978.67 a	11,098.73 ± 1545.02 b
$A_{90\_10}$	3583.82 ± 359.37 a	4322.64 ± 364.56 a	7597.90 ± 551.87 b
$∆t_{O\_90\_10}$	52.20 ± 1.96 a	59.94 ± 4.76 a	83.35 ± 7.88 b
$t_{O\_10}$	56.25 ± 2.28 a	64.31 ± 4.68 a	88.55 ± 7.74 b
$t_{A_{50}}$	27.95 ± 2.57 a	35.25 ± 5.95 ab	48.60 ± 6.88 b
$R_{max}$	5.65 ± 0.93 b	4.32 ± 0.37 ab	2.95 ± 0.58 a
	**GEw-A**	**GEw-B**	**GEw-C**
	**Chemical Parameters**		
Df-3-Gl (mg/L)	63.54 ± 0.29 c	55.47 ± 0.84 b	7.36 ± 0.04 a
Cn-3-Gl (mg/L)	11.85 ± 0.31 b	14.07 ± 0.16 c	1.39 ± 0.02 a
Pt-3-Gl (mg/L)	35.68 ± 0.27 c	29.81 ± 0.40 b	2.77 ± 0.02 a
Pn-3-Gl (mg/L)	32.73 ± 0.05 b	38.96 ± 0.74 c	10.90 ± 0.20 a
Mv-3-Gl (mg/L)	192.34 ± 0.21 c	181.83 ± 2.93 b	73.79 ± 1.25 a
IPT	37.75 ± 0.21 c	35.65 ± 0.00 b	19.13 ± 0.04 a
ABTS (mM)	19.82 ± 0.50 c	18.58 ± 0.13 b	11.02 ± 0.31 a
DPPH (mM)	11.91 ± 0.29 c	10.43 ± 0.07 b	7.05 ± 0.22 a
A420	3.98 ± 0.04 c	3.52 ± 0.08 b	1.59 ± 0.00 a
A520	6.28 ± 0.04 c	5.63 ± 0.07 b	2.02 ± 0.00 a
A620	1.20 ± 0.03 c	1.06 ± 0.04 b	0.38 ± 0.00 a
Color Intensity	11.47 ± 0.11 c	10.22 ± 0.19 b	3.98 ± 0.01 a

**∆*O_max_min_***: total oxygen consumed (hPa); ***O_min_***: minimum/final oxygen value (hPa); ***A_max_min_***: area under the oxygen consumption curve (hPa·h); ***O_mid_***: oxygen at half consumption time (hPa); ***O*_10_**: oxygen value that represents 10% of the range between the maximum and minimum values (hPa); **∆O_90_10_**:variation between oxygen 90% and oxygen 10% (hPa); **t_O_10_**: time when **O_10_** is reached (h); ∆**t**_O_90_10_: time variation between **t_0_90_** and **t_0_10_** (h); ***A*_90*_*10_**: area under the oxygen consumption curve and between **t_O_90_** and **t_O_10_** (hPa·h); ***R_max_***: maximum value of the oxygen consumption/rate curve (hPa/h); ***t_A_*_50_**: time when the area under the kinetic curve is half the total area under the curve (h). For the same row, different letters indicate significant differences among different GEws (A. B and C), according to Fisher´s LSD test (*p* < 0.05).
